# Impact of confinement in vehicle trunks on decomposition and entomological colonization of carcasses

**DOI:** 10.1371/journal.pone.0231207

**Published:** 2020-04-15

**Authors:** Stacey L. Malainey, Gail S. Anderson

**Affiliations:** Centre for Forensic Research, School of Criminology, Simon Fraser University, Burnaby, BC Canada; Animal Health Centre, CANADA

## Abstract

In order to investigate the impact of confinement in a car trunk on decomposition and insect colonization of carcasses, three freshly killed pig (*Sus scrofa domesticus* Erxleben) carcasses were placed individually in the trunks of older model cars and deployed in a forested area in the southwestern region of British Columbia, Canada, together with three freshly killed carcasses which were exposed in insect-accessible protective cages in the same forest. Decomposition rate and insect colonization of all carcasses were examined twice a week for four weeks. The exposed carcasses were colonized immediately by *Calliphora latifrons* Hough and *Calliphora vomitoria* (L.) followed by *Lucilia illustris* (Meigen), *Phormia regina* (Meigen) and *Protophormia terraenovae* (R.-D.) (Diptera: Calliphoridae). There was a delay of three to six days before the confined carcasses were colonized, first by *P*. *regina*, followed by *Pr*. *terraenovae*. These species represented the vast majority of blow fly species on the confined carcasses. Despite the delay in colonization, decomposition progressed much more rapidly in two of the confined carcasses in comparison with the exposed carcasses due to the greatly increased temperatures inside the vehicles, with the complete skeletonization of two of the confined carcasses ocurring between nine and 13 days after death. One confined carcass was an anomaly, attracting much fewer insects, supporting fewer larval calliphorids and decomposing much more slowly than other carcasses, despite similarly increased temperatures. It was later discovered that the vehicle in which this carcass was confined had a solid metal fire wall between the passenger area and the trunk, which served to reduce insect access and release of odors. These data may be extremely valuable when analyzing cadavers found inside vehicle trunks.

**Highlights**

- Confinement of pig carcasses inside car trunks in southwestern British Columbia, Canada delayed blow fly colonization by three to six days
- Temperatures inside cars were much higher than ambient temperatures and speeded up decomposition of the confined carcasses
- The presence of a metal fire wall between the passenger area and trunk of the car resulted in slowed decomposition and fewer insects colonizing.

## Introduction

Medico-legal or forensic entomology is commonly used in death investigations, primarily to estimate insect tenure on the remains to infer a minimum period of insect colonization, and hence a minimum post-mortem interval (_min_PMI). The first colonizers, primarily blow flies (Diptera: Calliphoridae), usually arrive immediately after death, and lay their eggs shortly afterwards, if conditions are suitable. Such suitable conditions include appropriate season, temperature, climatic conditions and, most importantly to this study, access [1]. Although blow flies are extremely adept at locating a carcass, often arriving within minutes of death [2–6], they need to be able to locate, then access the carrion. While blow flies can locate carcasses that have been wrapped [1] or confined [7] and will even lay eggs on suitcase zippers to allow the 1^st^ instar larvae to penetrate into the suitcase [8], there is a delay before they can gain access. Any barriers to insect colonization will increase the pre-colonization interval and thereby impact the estimation of insect tenure [9]. As well, concealing or confining remains has been shown to alter the normal composition, sequence and diversity of entomofauna which will impact the ability of a forensic entomologist to interpret the scene [2, 7, 10–12].

Some work has been done on the differences between bodies inside residences as compared with exposed outside, for example [2, 13, 14], but very little work has been done on bodies found inside vehicles, particularly those found in car trunks [15].

Although natural [16] suicidal [17, 18] accidental [19] and homicidal deaths [20] may occur inside vehicles, a car, and particularly a car trunk, is also a popular dumping site for a murdered body and usually involves placing the body in the trunk, then driving the vehicle into a rural area for dumping (Personal observation, GSA). The vehicle effectively provides a method of moving the body as well as concealment. On some occasions the vehicle and body may simply be left on a city street [1, 21].

As such bodies are not usually discovered immediately, by virtue of being concealed, forensic entomology is frequently involved in their analyses. It is well known that temperatures inside vehicles elevate very rapidly in sunlight and some work has shown differences between temperatures inside the trunk of a car versus the passenger area [22]. Work on carcasses inside passenger areas of cars in Australia has shown a delay in blow fly colonization of 24 to 28 h and an increase in speed of decomposition of three to four days, in comparison with exposed carcasses [15], however, there is a paucity of work in this area.

The objectives of this study were to determine the impact of concealing a body in the trunk of a car in comparison with open disposal of a body in a forest, on carcass decomposition and insect colonization. The study was designed to mimic a hypothetical homicide scenario in which a killer hides the body of their victim in the trunk of a car.

## Materials and methods

Six pig (*Sus scrofa domesticus* Erxleben) were utilized as human proxies. Three were placed in the trunks of vehicles placed in a forest and three were placed fully exposed, in cages, in the same forest for comparison.

### Research site

The research was conducted at the British Columbia Institute of Technology Research Forest in Maple Ridge, BC, Canada, approximately 45 km from Vancouver, BC. This woodlot is a 275 hectare parcel of crown forest land within the Coastal Western Hemlock Biogeoclimatic Zone, Dry Maritime Subzone [23]. It is dominated by western hemlock (*Tsuga heterophylla* (Raf. Sarg), western redcedar (*Thuja plicata* Donn *ex* D.Don) and Douglas fir (*Pseudotsuga menziesii* (Mirb) Franco) with smaller populations of other trees such as red alder *(Alnus rubras* Bong.), Sitka spruce (*Picea sitchensis (*Bong) Carr.) and grand fir (*Abies grandis* (Douglas) *ex* D. Don) Lindley) [23].

### Carcass placement

Six freshly killed pig carcasses were obtained and taken immediately to the research site. The pigs were killed with a single pin-gunshot to the head (this study was approved by Simon Fraser University Animal Care, Protocol Number 8051–06). The carcasses ranged from 25 to 32 kg. Each carcass was dressed in similar clothing: plaid boxer shorts, red t-shirts and cargo shorts.

Three of the carcasses were placed immediately into the trunks of three older vehicles (Pigs 1, 2 and 3) which were obtained from the Justice Institute of British Columbia (JIBC) Fire and Safety Division. Car #1 was a dark blue 1993 Mercury Topaz; Car #2 was a red/maroon 1991 Pontiac Bonneville, and Car #3 was a grey 1992 Pontiac Sunbird. Each vehicle was then placed at its research site. Car #1 was placed on an overgrown dirt path, in moderate to heavy shade, surrounded by trees on three sides (N 49 13.321’, W 122 26.628’). Car #2 was placed along a ditch beside a logging road and was heavily shaded. No GPS reading could be obtained due to canopy cover, but it was at least 1 km from Car #1. Car #3 was at the end of a logging road at the edge of a steep cliff (N 49 13.858’, W 122 25.515’). The road was more open so Car #3, although still in forest, was not shaded. It was situated approximately 1.8 km from Car #1. All cars were separated by at least 1 km.

The three remaining carcasses (Pigs 4, 5 and 6) were placed in the forest in dense shade similar to that of Car #2. Each carcass was placed inside a bear-proof cage (105 x 75 x 45 cm) constructed of rebar steel bars spaced approximately 10 cm apart to prevent large carnivore access but allow insect access. The sides of the cages were lined with a finer mesh to eliminate raccoons. The cage lids were hinged for ease of access. Cages were placed at least 100 to 200 m apart as recommended to reduce the chance of cross-contamination [24]. Recent studies have shown that this is adequate [25]. The cages themselves were located at least 1 km from the cars.

The day of placement was 24 July 2007 and was recorded as Day 0. The experiment ran for 29 days.

### Data collection

Smartbutton^®^ dataloggers, set to record temperature every hour, were placed with each pig to record microclimatic conditions. In the vehicles, a datalogger was placed beside the pig in the trunk and a second datalogger was placed in the passenger compartment of the car, hanging from the indicator.

Each carcass was examined twice a week. At each examination time, the carcasses were assessed, photographed, decomposition stage was noted, and a comprehensive insect sample was taken. Weather conditions at time of collection were noted and internal maggot mass temperatures were taken, when present. Insects were collected using standard forensic entomological collection techniques, with half of the immature samples being preserved immediately and the other half returned to the lab for rearing to adulthood.

Collection from the carcasses inside the vehicles was more complicated than that of the exposed carcasses as it was important to prevent any insect ingress or egress during collection. Therefore, prior to each opening of the car trunks, each car was covered with a large piece of clear plastic beginning at the rear windows and extending back several meters beyond the end of the car. The collector (SLM) then entered under the plastic and ensured it completely covered herself to the ground, before completing the assessment and collection. When collection was completed, the plastic was rolled up and secured on the top of the car, so as not to form an increased barrier to insect entry.

Insects were identified using Whitworth’s Keys to the Genera and Species of Blow Flies (Diptera: Calliphoridae) of America North of Mexico [26].

## Results

**Fig 1:**
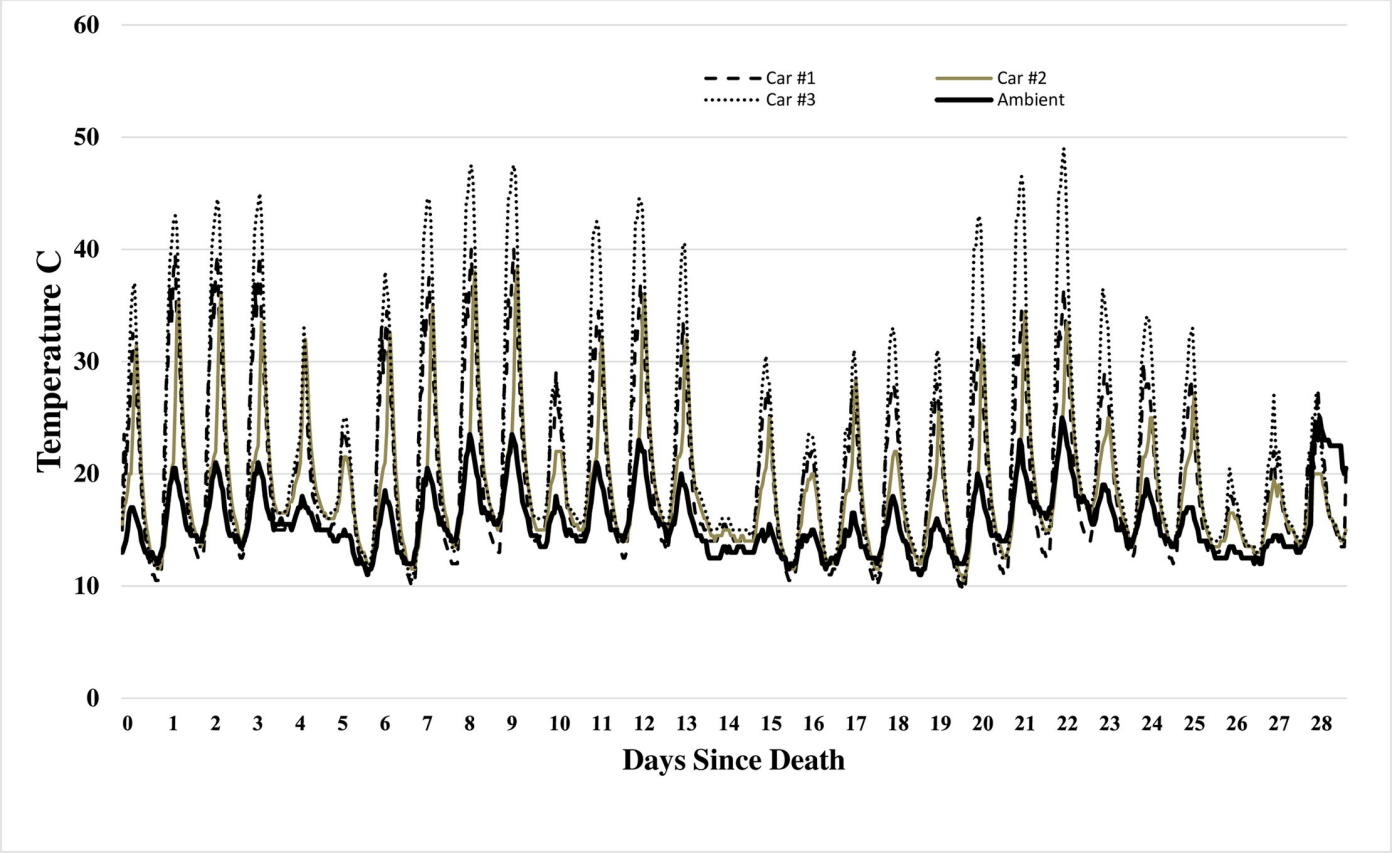
Temperature (°C) recorded in passenger area of all three cars compared with ambient temperature over duration of experiment.

Temperature was measured using dataloggers placed in the trunks and passenger areas of the cars and near the exposed carcasses to record ambient temperatures. Unfortunately, all three dataloggers placed inside the trunks of the cars failed. Fortunately, the loggers inside the passenger area functioned so a comparison could be made between external and internal temperatures. As expected, temperatures inside the vehicles were much greater than those outside during the day, with hourly temperatures ranging from 10 to 25°C higher inside the vehicles at peak temperatures (Fig 1). Car #1 and Car #2 exhibited similar internal temperatures with Car #3 frequently at least 5–10°C warmer than the other two vehicles (Fig 1). Night-time temperatures dropped to or even below ambient temperatures inside all the vehicles.

**Fig 2:**
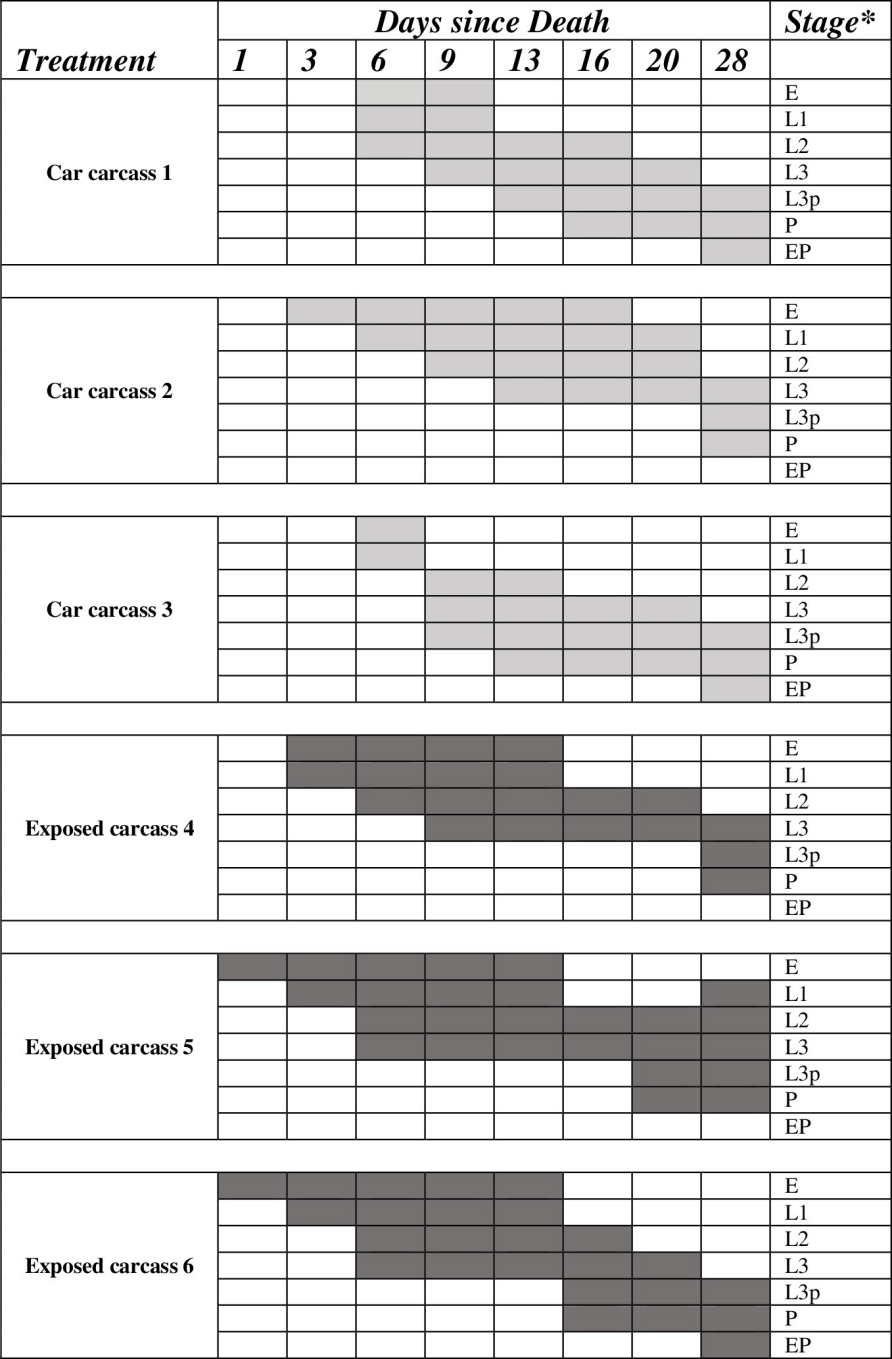
Developmental stages of blow flies (Diptera: Calliphoridae) colonizing exposed carcasses and carcasses inside vehicle trunks, over time.

The morning after placement, blow fly eggs (Diptera: Calliphoridae) were collected from two of the exposed carcasses (Pigs 5 and 6) (Table 1, Fig 2). Rearing showed that they had been laid on Day 0. No eggs were noted on the third exposed carcass but there was noticeable adult blow fly activity. Unfortunately, the subsequent larvae did not complete development so were not identified. Inside the car trunks, no flies had yet accessed any of the carcasses and the carcasses were still in rigor mortis.

**Table 1:** Comparison of Calliphoridae fauna colonizing pig carcasses inside vehicle trunks *versus* exposed carcasses.

	Carcasses in Car Trunks			Exposed carcasses		
* *	*Pig #1*, *Car #1*	*Pig #2*, *Car #2*	*Pig #3*, *Car #3*	*Carcass #4*	*Carcass # 5*	*Carcass #6*
**Day 1**	Rigor mortis still present No insects	Rigor mortis still present No insects	Rigor mortis still present No insects	Some greenish skin discoloration, rigor mortis still present.No insects	Some greenish skin discoloration, rigor mortis still present. Calliphoridae Eggs	Some greenish skin discoloration, rigor mortis still present. Calliphoridae Eggs
**Day 3**	Early bloat, green discoloration at stomach No insects	Early bloat Eggs *Phormia regina*	Full bloat No insects	Slight bloat Eggs, L1 *Calliphora latifrons Lucilia illustris*	Slight bloat Eggs, L1 *Calliphora latifrons L*. *illustris*	Slight bloat Eggs, L1 *Calliphora vomitoria*
**Day 6**	Full bloat, putrefaction, start of active decay Eggs, L1, L2 *P*. *regina Protophormia terraenovae L*. *illustris L*. *sericata*	Full bloat, putrefaction, start of active decay Eggs, L1 *P*. *regina Pr*. *Terraenovae*	Full bloat, putrefaction, start of active decay Eggs, L1, MM *P*. *regina Pr*. *Terraenovae*	Bloat, marbling Eggs, L1, L2, MM (17°C) *C*. *latifrons*	Bloat, marbling Eggs, L1, L2, L3 MM (18°C) *P*. *regina*	Bloat, marbling, start of active decay Eggs, L1, L2, L3 MM (25°C) *C*. *vomitoria L*. *illustris*
**Day 9**	Active decay Eggs, L1, L2, L3, MM (32°C) *P*. *regina Pr*. *Terraenovae*	Full bloat Eggs, L1, L2 *P*. *regina Pr*. *Terraenovae*	Advanced decay, skeletonization L2, L3, L3p, MM (39°C) *P*. *regina Pr*. *Terraenovae*	Active decay Eggs, L1, L2, L3, MM *C*. *latifrons L*. *illustris*	Active decay Eggs, L1, L2, L3, MM *C*. *vomitoria P*. *regina Pr*. *Terraenovae L*. *illustris*	Active decay Eggs, L1, L2, L3, MM *C*. *vomitoria*
**Day 13**	Skeletonized L2, L3, L3p, MM (32°C) *P*. *regina Pr*. *terraenovae*	Bloat Eggs, L1, L2, L3 *P*. *regina Pr*. *terraenovae*	Skeletonized L2, L3, L3p, P *P*. *regina Pr*. *terraenovae*	Active decay Eggs, L1, L2, L3, MM (31°C) *C*. *vomitoria L*. *illustris P*. *regina*	Active decay Eggs, L1, L2, L3, MM (38°C) *C*. *vomitoria L*. *illustris*	Active decay Eggs, L1, L2, L3 (42°C) *C*. *vomitoria L*. *illustris P*. *regina*
**Day 16**	Skeletonized L2, L3, L3p, P *P*. *regina Pr*. *terraenovae*	Active decay Eggs, L1, L2, L3, MM *P*. *regina Pr*. *terraenovae*	Skeletonized L3, L3p, P *P*. *regina Pr*. *terraenovae*	Advanced decay L2, L3, MM (28°C) *C*. *vomitoria P*. *regina*	Advanced decay L2, L3, MM (40°C) *C*. *vomitoria P*. *regina Pr*. *terraenovae*	Advanced decay L3, P *P*. *regina*
**Day 20**	Skeletonized L3, L3p, P *P*. *regina Pr*. *terraenovae*	Active decay L1, L2, L3 *P*. *regina Pr*. *terraenovae*	Skeletonized L3, P *P*. *regina Pr*. *terraenovae*	Advanced decay L2, L3 *P*. *regina*	Advanced decay L2, L3, L3p, P *P*. *regina*	Mostly skeletonized L3, P *P*. *regina Pr*. *terraenovae*
**Day 28**	Skeletonized L3p, P, EP *P*. *regina Pr*. *terraenovae*	Active decay L3, L3p, P *P*. *regina Pr*. *terraenovae*	Skeletonized L3p, P, EP *P*. *regina Pr*. *terraenovae*	Advanced decay L3, L3p, P	Advanced decay L1, L2, L3, P *P*. *regina Pr*. *terraenovae*	Skeletonized L3p, P, EP *P*. *regina*

L1 = 1^st^ instar, L2 = 2^nd^ instar, L3 = 3^rd^ instar, L3p = Prepuparial 3^rd^ instar, P = live puparia, MM = maggot masses, EP = empty puparia

By Day 3, all three exposed carcasses were slightly bloated. Pig 4 had eggs along the back of the head, clothing and under the mandible and 1^st^ instar larvae were crawling in the mouth. Pig 5 had larvae in the nostrils and one orbit. Of the three, Pig 6 had much greater activity, with larvae in the mouth and the underside of the face. All three exposed carcasses were colonized by *Calliphora* sp. (*Calliphora vomitoria* (L.) and *C*. *latifrons* Hough), but Pigs 4 and 5 were also colonized by *Lucilia illustris* (Meigen). Inside the car trunks, a strong odor of putrefaction was noted, and adult blow flies were seen around the outside of the cars, sometimes in large numbers, as well as an adult Silphidae (Coleoptera) although Calliphoridae eggs (*Phormia regina* (Meigen)) were only found on one carcass (Table 1). Car #3 was in full sunlight and was covered in many more adult blow flies and a few flies had entered the passenger area of the car, but none had entered the trunk. All the car trunk carcasses were in bloat and were more bloated than the exposed carcasses.

**Fig 3:**
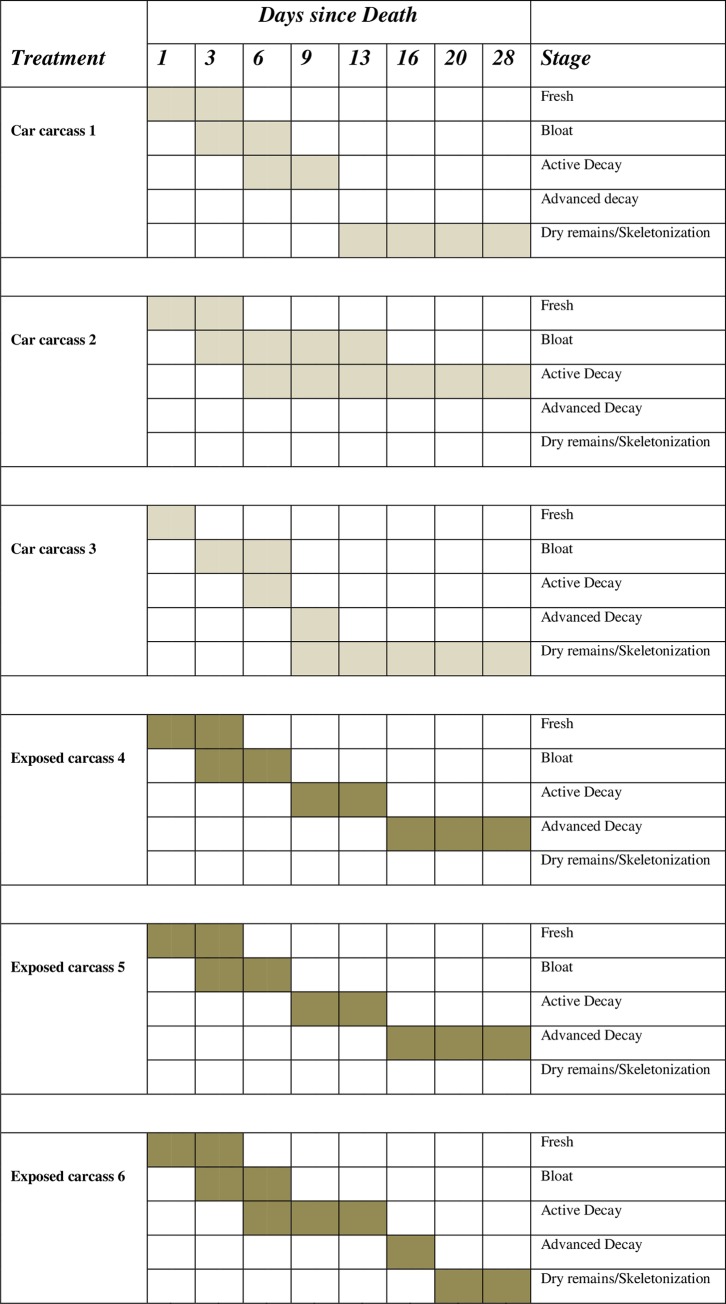
Decompositional stages of carcasses inside vehicle trunks and exposed carcasses, over time.

By Day 6, 3^rd^ instar larvae were found on exposed Pigs 5 and 6, and visible maggot masses were noted on all three exposed carcasses (Table 1, Fig 2). The masses on Pigs 4 and 5 were quite small, with internal temperatures similar to that of ambient (17–18°C), but the mass on Pig 6 covered most of the mouth, nose, eyes, ears, and back of head, with internal temperatures of 25°C. As well as the species noted before, *P*. *regina* was also noted on Pig 5. All exposed pigs were bloated and marbled, with Pig 6 beginning to enter active decay. At the same time, carcasses inside the car trunks had greatly deteriorated, with a strong odor noticeable up to 3 m away; fluid had been expelled and they were in full bloat with putrefaction and active decay evident (Fig 3). All three car trunk carcasses had been colonized by blow flies with 1^st^ instar larvae found on all and 2^nd^ instar larvae on Pig 1. Approximately five to ten adult blow flies had entered the trunk of Car #1 and an adult *Lucilia* sp. was noted in the mouth. Larval rearings from this carcass included *P*. *regina* and *Protophormia terraenovae* (R.-D.), together with *Lucilia illustris* and *Lucilia sericata* (Meigen), with the majority being *P*. *regina* throughout the experiment. The other two confined carcasses only supported the two Chrysomyinae and no further Luciliinae were collected on any of the confined carcasses at any other date. Pig 3, located in the least shaded area, had the most insect activity with over 100 adult blow flies inside the passenger area of the car and a large mass of 1^st^ instar larvae on the face.

**Fig 4:**
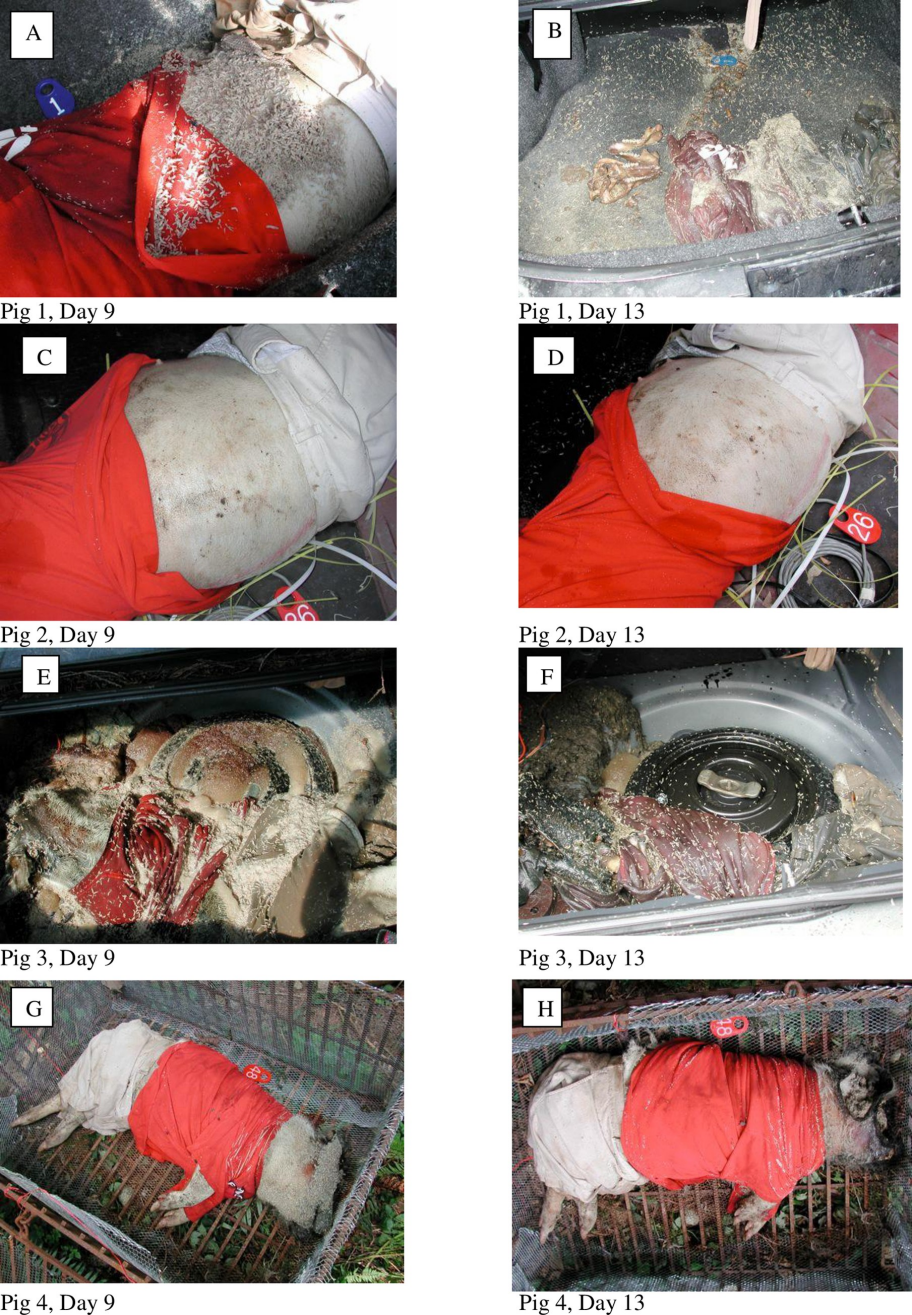
Comparison of decompositional changes between Days 9 and 13. A and B, Pig 1 in Car #1 on Days 9 and 13. C and D, Pig 2 in Car #2 on Days 9 and 13, E and F, Pig 3 in Car #3, on Days 9 and 13 and G and H, Pig 4, exposed, on Days 9 and 13.

Third instar larvae were not collected from exposed Pig 4 until Day 9, and the majority of larvae from all three exposed carcasses were still in the second instar, with some 3^rd^ instar present (Table 1, Fig 2). The exposed carcasses were primarily colonized by *C*. *vomitoria*, *P*. *regina*, *L*. *illustris* with *Pr*. *terraenovae* found on Pig 5 by Day 9 and all were in active decay (Figs 3 and 4). At the same time, greater changes had taken place inside the trunks of the cars. The face of Pig 1 had been skeletonized and the rest of the carcass was in active decay with two maggot masses present, and larvae ranging from 1^st^ to 3^rd^ instar, although the majority were in the 1^st^ instar. Approximately 50 adult blow flies were found in the passenger area of the car. Pig 2 was still in bloat and had only a few adult blow flies in the passenger area and small aggregations of 1^st^ and 2^nd^ instar larvae were present (Fig 4). Pig 3, however, had a dramatically different appearance which was noted at first approach to the car. From the outside of the vehicle, prepuparial or post-feeding 3^rd^ instar blow fly larvae were seen inside the passenger area of the vehicle as far up as the windshield and were seen coming out of the closed trunk. Opening the trunk revealed that much of the soft tissue of the carcass had been removed, with some maggot masses still present. The entire inner surface of the trunk was covered in vast numbers of prepuparial 3^rd^ instar larvae, with some dead adult flies present in the passenger area (Fig 4).

By Day 13, the majority of larvae on the exposed carcasses were in the 3^rd^ instar (Fig 2). The carcasses continued to be dominated by blow fly larvae as before but, for the first time, adult Coleoptera (Silphidae) were noted on Pig 4. Maggot mass temperatures on all three exposed pigs were between 11 and 22°C higher than the ambient temperature of 20°C (Table 1). Both Pigs 5 and 6 supported large maggot masses and remained in active decay (Table 1, Fig 3). Inside the vehicles, Pigs 1 and 3 were completely skeletonized (Figs 3 and 4). The bones of Pig 1 were scattered towards the back of the trunk and prepuparial larvae were seen crawling in and outside the trunk, and along the rear window. A maggot mass was still present. Many dead adult blow flies were seen inside Car #3 along the rear window and pooled in the seats, and living puparia were collected for the first time, both in the trunk and in the body of the car, burrowed into the front drivers side floor carpet. Interestingly, Pig 2 was quite different from the other confined pigs, as it was intact and still bloated, with much fewer larvae present and no maggot masses (Figs 3 and 4).

By Day 16, living puparia were first observed on one of the exposed carcasses (Pig 6) and maggot masses continued to be at a considerably higher temperature than ambient (10–18°C) (Table 1). The stomach area of Pig 4 was covered in mites, with blow fly larvae localized at the head area. Much of the skin had dried and the carcasses were in advanced decay (Fig 3). When Car #1 was approached, many prepuparial larvae were noted outside the car, crawling along the rear bumper, inside the passenger area of the car and along the rear window, as well as dead adult flies. Living puparia were found inside the trunk and passenger area, mostly in the carpeted areas of the driver and front passengers areas as well as the rear passenger carpet, as well as 2^nd^ and 3^rd^ instar larvae, possibly searching for a further source of food. No masses were present. Pig 3 was similar in that large numbers of living puparia were found in all carpeted areas of the passenger area of the car although no empty puparia were found. Prepuparial larvae were seen exiting the trunk of the car and the soft tissue of Pig 3 had been entirely removed. Pig 2 had finally entered active decay with a small maggot mass and all larvae feeding, but the number of larvae was much fewer than on the other two confined carcasses. No maggots were found in the passenger area. Fresh eggs and 1^st^ instar larvae were found on the carcass. Mites were also present.

By Day 20, *P*. *regina* living puparia were found on two of the exposed carcasses (5 and 6) and prepuparial larvae were seen in the surrounding areas. The bulk of the soft tissue had been removed from Pig 6 by this time and many mites were present. In the car trunks, large numbers of living puparia were found in the trunks and passenger areas of Cars #1 and #3, with a few prepuparial 3^rd^ instar larvae still present, whereas Pig 2 still only supported feeding larval blow flies.

By the final day of collection, Day 28, blow fly living puparia were present on all three exposed pigs (Table 1). The first empty puparia was found on Pig 6. Piophilidae adults were collected from Pigs 4 and 5 and, interestingly, an empty Piophilidae puparial casing was located on Pig 5, indicating that Piophilidae larvae must have been missed earlier. Thousands of live and dead blow flies were present inside Cars #1 and #3, presumably those that had emerged from living puparia. Many living puparia and empty puparia were present, three to four layers deep in some cases. Although living puparia and empty puparia were found in the trunks of these cars, the vast majority had moved into the passenger area of the car to pupate. Living puparia were finally found associated with Pig 2, although a maggot mass was still present. Interestingly, Piophilidae living puparia were also found in and along the rim of the trunk.

The experiment was terminated at Day 28.

## Discussion

Pig carcasses are a commonly-used human proxy in forensic entomology research. They are omnivorous so have similar gut fauna, are relatively hairless and have similar skin [27]. Ideally, human cadavers would be best for such studies but although more human body research facilities are being developed in the US and worldwide recently, they are still limited, with restricted cadaver replication, due to cadaver availability and differences between human cadavers. Relatively uniform pig carcasses can be easily obtained and do not require the same amount of security for placement. Some studies have shown differences between pig and decomposition rates in some areas [28], but so far, no differences have been seen between insect species collected from pig and human cadavers [3, 29, 30] and insect development is dependent on temperature rather than carcass type. A recent review has shown that using pig carcasses as human proxies is much more practical than using human cadavers as it makes controlling for confounding parameters much easier and increases the ability to replicate carcasses [31].

### Temperature

It is extremely unfortunate that all the trunk dataloggers failed. Although no faults could be found and the upper limit for the loggers was 85°C, so should not have been surpassed, all three failed. Therefore, trunk temperatures could only be surmised based on internal car temperatures recorded in the passenger area of the car. Experiments inside vehicles in Western Australia showed that trunk temperatures were usually 10°C higher than ambient temperatures whereas passenger area temperatures could be as much as 20–30°C higher than ambient, reaching almost 70°C, on days with ambient temperatures near 40°C [32]. It was also noted that the internal temperatures within a dark car were almost 5°C higher than a white car under identical hot summer daytime conditions [32]. The present experiments were conducted under the much cooler summertime temperatures of southwestern British Columbia, with ambient temperatures very rarely above 25°C, dropping to as low as almost 10°C on some nights (Fig 1). Nevertheless, greatly increased temperatures were seen inside the vehicles in comparison with ambient temperatures. Although we attempted to acquire vehicles which were similar, we were, by necessity, dependent on receiving vehicles which were considered relatively valueless, so we were not able to obtain identical vehicles or colors. Similarly, because of the experimental type, research site and the need for separation of experimental sites to avoid cross contamination, we were unable to choose identical placement sites for each vehicle. Consequently, Car #1 was dark blue and placed in moderate to heavy shade, Car #2 was red/maroon and placed in heavy shade and Car #3 was grey and placed in sunlight. Daily car temperatures were dramatically higher than ambient with, as would be expected, Car #1 and Car #2, in shade, being 5–10°C cooler than Car #3, in the sun, with temperatures reaching almost 50°C in Car #3 at one point. This was reflected in the much more rapid skeletonization of Pig 3 in Car #3 (9–13 days) and the more rapid insect development. Based on the Australian experiments, it is probable that temperatures within the car trunks were not as high as in the passenger area but would still have been greatly elevated above ambient. Night-time temperatures dropped to or below ambient temperatures. In a homicide case in a metropolitan area in a city in the same biogeoclimatic zone, human remains were found under a blanket in the trunk area of a hatchback vehicle [1]. In order to estimate the temperatures inside the vehicle for the duration of insect development, a vehicle of similar age, type, and color was placed at the crime scene once the crime vehicle was removed. A SmartReader 1^®^ datalogger, identical to those used in the present experiment, was placed under a similar blanket in the trunk region to record temperatures for the subsequent ten days which were then compared using a regression analysis with the nearest Environment Canada weather station. The analysis showed a good correlation between the car trunk temperatures and the government weather station, generating an equation that could then be used to estimate car trunk temperatures. The internal trunk temperatures ranged from 10–15°C above the ambient temperature but dropped to or below ambient at night, as it did in this study. These data were used to predict the car temperatures and estimate the age of the oldest stage of insects on the remains [1].

### Delay in insect colonization related to confinement

The exposed carcasses were colonized rapidly after placement, with blow fly eggs being laid on two of three carcasses within hours of placement. This was expected as blow flies are usually the first taxon to arrive on a cadaver and are extremely efficient at locating odor plumes immediately after death [33].

Colonization was delayed for three days up to as much as six days by confinement inside a car trunk. Although blow flies have evolved very specialized abilities to locate odors, such as the volatile organic compounds (VOCs) released from a cadaver, their ability to locate the odor source may be limited when the cadaver is protected or packaged in some way. Similarly, once located, time to colonization may be increased if access to the remains is barred in some manner. In a house in Alberta, colonization of pig carcasses was delayed by five days [2] and in Hawai’i, wrapping a carcass, in order to simulate an actual homicide, delayed blow fly oviposition by 2.5 days [34]. In Malaysia, wrapping monkey carcasses delayed colonization by one to 13 days depending on insect species [35]. In Australia, blow fly attendance at pig carcasses placed in the passenger area of vehicles was delayed by 16–18 h and oviposition was delayed until 24–28 h after death [15]. The greater delay seen in our study may have been due to the fact that the remains were further confined inside the trunk of the vehicles rather than the passenger area as increased concealment increases the length of time of delay. In a study of carcasses in car trunks in Louisiana, a delay of three days was noted [27] and in a study of confined carcasses in North Carolina, baby pig carcasses were concealed in mock attics, with level of concealment ranging from minimal (placed on the floor of the attic), moderate (wrapped in a blanket in an attic) and well-concealed (placed in a Rubbermaid^®^ bin with lid, in an attic) and compared. Delay in colonization ranged from 35 to 768 h based on level of concealment [7]. In the UK, a delay of one to three days was noted when a pig part was placed inside a suitcase [8]. Amount of concealment has also been shown to impact the patterns of Coleoptera arrival and tenure on carcasses concealed in garbage cans, drums and suitcases [36]. Very few Coleoptera were noted on exposed or confined carcasses in this study.

In these experiments, the cars were sealed, with all windows and doors closed, yet blow flies were still able to detect and enter the vehicles. In four homicides in Chicago, IL, in which the bodies were concealed in car trunks, *P*. *regina*, *L*. *sericata* as well as *Cochliomyia macellaria* (F.) larvae were collected from the remains. Again, all doors and windows were sealed, the only entrance, a drainage hole under the spare tire, yet the insects were able to gain access [21].

The blow flies did not appear to be deterred from laying eggs on the carcasses in the trunks by darkness. Many studies from around the world have shown that blow flies almost never oviposit during the night-time (for example, [37–40]), but this is a true diurnal rhythm and not related to light levels. In fact, blow flies are known to lay eggs in the dark, for example, in dark places including basements, cellars and chimneys [41], caves [42, 43] and car trunks [1]. Indeed, blow flies can often be induced to oviposit in laboratory conditions by placing bait inside an old fashioned dark film canister (Byrd, J. Pers. Comm.). Therefore, darkness would be unlikely to hinder oviposition.

### Insect colonization patterns

The insect colonization of carcasses that are concealed or confined has been shown to be different from those colonizing carcasses exposed outdoors in many areas of the world [2, 7, 12, 14, 35, 36, 44]. In the present study, *P*. *regina* was the first blow fly species to colonize the confined carcasses, followed by *Pr*. *terraenovae* which together dominated the vehicular carcasses, although *P*. *regina* was the most numerous species in the car trunk carcasses by far, and on one occasion, on one confined carcass two Luciliinae were collected. The species composition of the exposed carcasses was very different, with *C*. *latifrons* and *C*. *vomitoria* colonizing first, followed by *L*. *illustris*, *P*. *regina* and *Pr*. *terraenovae*. Greater species diversity was seen on the exposed carcasses, as has been noted on carrion outdoors [2] as well as in other experiments within vehicles although in Australia, diversity matched that on exposed carcasses by the later stages of decomposition [15].

The presence of clothing can impact the rate of decomposition and insect colonization patterns as clothing affects the temperature and humidity of the carcass, provides protection for insects, increases the carcasses’ moisture, allowing them to feed on the skin, and provides more oviposition sites [45–48]. Some studies have indicated differences in rate of decomposition between clothed and unclothed carcasses [46, 49], whereas others have found little to no effect [50, 51]. In some cases, clothing kept carcasses moist, prolonging the later decay stages, although insect arrival times and tenure were similar in both clothed and unclothed carcasses, with a few exceptions [49]. In Australia it was noted that maggot masses remained for longer on clothed carcasses [49] and in South Africa, clothing facilitated maggot movement, impacting maggot mass distribution [47]. Such differences between clothed and unclothed carcasses has also been shown to be seasonal in some geographical areas [52]. In this experiment, the carcasses were all clothed to mimic human homicides as casework has shown that the majority of homicide victims are clothed, or wrapped in cloth (Personal observations, GSA).

The colonization of all carcasses was dominated by blow flies, although a few adult Coleoptera did attend and Piophilidae were attracted to both sets of carcasses. Similarly, in a human case, a cadaver was found under a blanket in the trunk area of a hatchback car, parked on a city street. The car had several severely rusted areas and the front window was open slightly. As in the present study, the remains had been colonized primarily by *P*. *regina* and *Pr*. *terraenovae*, although a few younger larvae of *C*. *vomitoria* were collected. Hymenopteran parasites had also been able to enter the vehicle and parasitize some living puparia [1]. Interestingly, under the rear of the car, a drainage area had allowed decompositional material to run out and a secondary colonization, with much greater species diversity, had occurred including the same blow fly species as well as *C*. *latifrons* and Muscidae larvae (*Hydrotaea* sp.) and various Coleoptera, including Nitidulidae, Staphylinidae and Tenebrionidae [1].

*Calliphora* spp. were entirely absent from the confined carcasses, and although a single adult *Lucilia* sp. was observed in the mouth of one of the confined carcasses, only three specimens were raised and only from a single occasion. *Calliphora* spp. tend to prefer cooler temperatures [42, 53] and can be active as low as 1.7 ^o^C [54], which might explain why *Calliphora* spp. did not colonize the carcasses in the car trunks, as the extreme heat may have repelled adults, or killed eggs. This does not explain the lack of *Lucilia* spp. as these tend to prefer warmer temperatures [42]. The more commonly found rural species, *L*. *illustris*, however, is not usually found indoors [55] so it may be less attracted to carrion that is sequestered in any manner. The presence of *L*. *sericata* is somewhat unusual, as although *L*. *illustris* is considered a rural blow fly, *L*. *sericata* is usually considered urban [55]. Interestingly, in earlier research in this same forest, approximately 12 km distant from the present sites, only typically rural species were collected from extensive carrion experiments, including *C*. *vomitoria*, *L*. *illustris*, and the more ubiquitous species, *P*. *regina* and *Pr*. *terraenovae* [45]. The earlier experiments were conducted in deeper forest than the present experiments, further from roads and with much less human activity, and the present experiments were conducted in a woodlot that did allow public access, albeit not by motorized vehicles, which may mean that human refuse and activity has increased the presence of synanthropic species in this area of the forest. As well, only two specimens of *L*. *sericata* were raised so few conclusions can be drawn. *Calliphora latifrons* is a species that has been shown to be reluctant to enter homes [2] so may be also less attracted to confined remains.

Very large numbers of blow fly larvae were seen on two of the three confined pigs once active decay began; much greater than those seen on the exposed carcasses, suggesting that although the presence of the car may have originally acted as a barrier to the release of VOCs at first, once decomposition advanced, the increased temperatures inside the vehicle increased the release of VOCs and attracted more blow flies. This is different from that seen in a comparison of decomposition inside and outside a house, in which colonization was much slower with much fewer insects inside the house compared with outside [2]. Alternatively, the exposed carcasses may have been more exposed to predation and parasitism, as well as adverse weather conditions such as desiccation and drowning, than those protected inside the vehicles.

Prepuparial 3rd instar blow fly larvae leave the food source to seek a suitable site for metamorphosis. The distance larvae disperse varies with species and substrate as well as other biotic and abiotic factors [2]. Some species, such as *Pr*. *terraenovae*, prefer to pupariate close to the carcass [41], whereas others, such as *L*. *sericata* disperse over much greater distances [56]. Dispersal was not limited in the exposed carcasses but most of the prepuparial larvae inside the vehicles were unable to escape their confines. In most cases, living puparia were found at the farthest possible point from the corpse, embedded in the carpet of the drivers’ side seat. This suggests that the larvae were actually capable of dispersing farther but were unable to do so because of their inability to exit the vehicle. This is corroborated by the plethora of dead flies that gradually accumulated in the body of the vehicles as the experiment progressed. Indeed, it was increasingly evident that calliphorids were able to enter the vehicle and oviposit on the carcass yet lacked the capacity to exit.

Some insects show a preference for carrion in sunlit areas versus shaded areas and vice versa, although this seems to vary geographically [1, 57]. Sunny areas are obviously going to be warmer than shaded areas and this speeds up decomposition [58, 59], but it also has been shown to increase the diversity of blow fly species which colonize. In Alberta, decomposition rate was not affected by habitat but did impact species abundance [60] and in Saskatchewan a greater diversity of species was seen on sunlit carcasses [6]. Although Car #3 was in sunlight in the present study and the other two cars were in shade, this had no impact on the species that colonized the remains, with the same species being present on all car trunk carcasses. However, the placement of Car #3 in the sun had a major impact on the internal car temperatures which were regularly 5–10°C higher than Cars #1 and #2. This was reflected in the much faster decomposition of Pig 3 than the other confined carcasses. As well, insect development was faster on Pig 3 than the other carcasses.

Overall, blow fly development was much more rapid inside the car trunks than outside (with the exception of Pig 2, discussed below), despite the three to six day delay in colonization, due to the much higher car temperatures. This is very important to understand in a forensic investigation involving cadavers inside cars. Firstly, it can be expected that there will be a delay in insect access to the cadaver and colonization may be delayed for several days. This needs to be taken into account when analyzing cases of body concealment in vehicles and emphasizes the need to provide a _min_PMI rather than attempt to provide a maximum. When the body is confined, as in these simulations, it is best to indicate a _min_PMI based on the insect age together with the caveat that insect colonization was most probably delayed by three to six days so death may have occurred three to six days or more earlier than the given _min_PMI. Secondly, once colonized, insect development will be much more rapid inside a vehicle compared with normal exposure. This is more difficult to quantify but experiments have suggested the differences between trunk and ambient temperatures [32] which could allow careful extrapolation from weather station data to trunk temperatures. If these two factors are not considered, then the entomological estimation of age of insects and consequent inference of estimation of minimum elapsed time since death may be inaccurate by as much as several days.

### Later colonizers

This experiment ran for only 29 days so the primary colonizers of both sets of carcasses were early colonizers, almost entirely comprised of Calliphoridae, although a very few Piophilidae did colonize. Piophilidae are usually later colonizers, although larvae of other later colonizers, *Musca domestica* L. (Diptera: Muscidae) and *Megaselia scalaris* Loew (Diptera: Phoridae) have also been reported from a body inside a vehicle ten days after death in Saudi Arabia, with the usually expected Calliphoridae being absent [16]. This again highlights the differences that may be seen between insects colonizing exposed carcasses in comparison with those in more confined areas. Phoridae, for example, are usually later colonizers of exposed remains [61] but may be the first colonizers on indoor cadavers [14, 62]. It appears some members of this family actively seek out more concealed areas, as is famously illustrated by *Conicera tibialis* Schmitz otherwise referred to as the ‘coffin fly’ which is known to burrow down to buried remains and colonize for many generations, being found up to 18 years after death [63]. Some of the usual later successional species have often been more limited on bodies found indoors [2] although some species easily access indoor remains. In a case in BC, the remains of a man last seen alive the previous December were found in the trunk of a vehicle on an urban street approximately 10 months later [1]. The body had mummified over the winter period and had been colonized by calliphorids (*L*. *sericata* and *P*. *regina)* as well as larval Sarcophagidae, empty puparia and larvae of two species of Piophilidae and several families of Coleoptera, including Dermestidae, Cleridae and Nitidulidae [1]. The wide range of species that entered the vehicle showed that the vehicle itself, although probably delaying colonization, did not provide a major barrier over time.

### Decomposition

The increased temperatures inside the vehicles greatly speeded up the decomposition of the carcasses inside the car trunks with complete skeletonization occurring on carcasses inside vehicles within two weeks, while the exposed carcasses were still in active decay. This is also much more rapid than has been seen in other decomposition experiments conducted in the same general region [3, 64]. In Australia, pig carcasses in the passenger area of cars progressed through the decomposition stages three to four days faster than those exposed outside, due to the higher vehicular temperatures, again despite an initial delay in colonization [15].

The carcass in Car #2 decomposed and was colonized in an entirely different manner than the other carcasses. Consistently throughout collection, fewer maggots were present on this pig compared with all the other pigs (both confined and exposed). Decomposition was dramatically slower, with the carcass appearing to mummify rather than being consumed by insects, despite the increased temperatures in this vehicle being very similar to Car #2 which was also in shade. For reasons unbeknownst during the collection phase, Pig 2 was an anomaly. At the time of this experiment, the reason for this was unclear, but was revealed in a later experiment in which all three cars were burned in simulated arsons [65]. At that point, it was discovered that Car #2 alone had a solid metal fire wall inserted between the trunk and the passenger compartment in order to protect passengers from a fire in the trunk area. Although Pig 2 was the first to be colonized, it was only very lightly colonized throughout and never attracted the vast numbers of blow flies seen on Pigs 1 and 3.

Several mechanisms could account for the influence of the metal fire wall. This could have operated as an impediment to insect access. Carrion protected from insects decays much more slowly when compared with carrion to which insects have access [11, 66, 67] and a different set of decomposition stages has been proposed for carcasses which are not colonized [68]. Insects are primarily attracted to carrion by the VOCs released after death [69] and the presence of the metal wall may have affected the release of odor plumes, thereby reducing the sphere of influence for this carcass. In other words, perhaps only blow flies that happened to be close to this vehicle could pick up the odor plume, whereas the release of VOCs was much greater for the other carcasses. This would result in greater numbers of flies being attracted to these bodies, thus hastening decomposition. Although only a single case, the differences between the colonization and decomposition of Pig 2 and Pigs 1 and 3 strongly suggest that the presence of the fire wall greatly impacted the decomposition of this carcass. This was not a feature that was visible to us at the time of this experiment and was only discovered once the car was burned. Therefore, it will be necessary to examine vehicle specifications in order to determine whether a fire wall is present.

## Conclusions

Carcasses confined in car trunks were not colonized by blow flies for between three and six days after placement in comparison with carcasses directly exposed, which were colonized immediately. This is important to consider when analyzing forensic cases in which cadavers in fresh and bloat stages are discovered. However, greatly increased temperatures within vehicles rapidly speeded up insect development after this point, resulting in much more rapid progression through decompositional stages. Although resulting in a delay in colonization, the vehicle itself was easily accessible to insects once the odors released by the cadavers increased after a few days. The major exception to this was in a vehicle in which a fire wall was present between the passenger area and the trunk. In that case, very few insects either detected the carrion, or were able to penetrate the trunk resulting in much slower decomposition and much lower abundance of insects, although species diversity did not change. This resulted in the carcass inside a car with a fire wall decomposing at a slower rate even than the exposed carcasses. These are all factors that must be considered when analyzing remains found inside the trunk of vehicles.
